# A new building block for DNA network formation by self-assembly and polymerase chain reaction

**DOI:** 10.3762/bjoc.10.104

**Published:** 2014-05-07

**Authors:** Holger Bußkamp, Sascha Keller, Marta Robotta, Malte Drescher, Andreas Marx

**Affiliations:** 1 Department of Chemistry and Konstanz Research School Chemical Biology, University of Konstanz, Universitätsstraße 10, 78457 Konstanz, Germany

**Keywords:** AFM, branched DNA, DNA, DNA polymerase, nanotechnology, nucleic acids, PCR, self-assembly

## Abstract

The predictability of DNA self-assembly is exploited in many nanotechnological approaches. Inspired by naturally existing self-assembled DNA architectures, branched DNA has been developed that allows self-assembly to predesigned architectures with dimensions on the nanometer scale. DNA is an attractive material for generation of nanostructures due to a plethora of enzymes which modify DNA with high accuracy, providing a toolbox for many different manipulations to construct nanometer scaled objects. We present a straightforward synthesis of a rigid DNA branching building block successfully used for the generation of DNA networks by self-assembly and network formation by enzymatic DNA synthesis. The Y-shaped 3-armed DNA construct, bearing 3 primer strands is accepted by *Taq* DNA polymerase. The enzyme uses each arm as primer strand and incorporates the branched construct into large assemblies during PCR. The networks were investigated by agarose gel electrophoresis, atomic force microscopy, dynamic light scattering, and electron paramagnetic resonance spectroscopy. The findings indicate that rather rigid DNA networks were formed. This presents a new bottom-up approach for DNA material formation and might find applications like in the generation of functional hydrogels.

## Introduction

DNA has found applications in the field of nanotechnology due to its inherent properties. The simplicity and predictability of DNA secondary structure are of outstanding potential for the design of self-assembled architectures [1–3]. Inspired by naturally existing self-assembled DNA architectures known as Holliday junctions, Seeman envisioned the approach to organize DNA with branched DNA (bDNA) and thereby initiated the field of structural DNA nanotechnology [4]. Since then several reports have described the generation of bDNAs self-assembling to predesigned architectures with dimensions on the nanometer scale [5–9]. Based on this, numerous examples of 2D arrays [10–14], DNA origami [15] and complex 3D DNA nanostructures [16–20] were generated by intelligent algorithmic assembly design strategies [5–9]. Besides, the directed assembly of cells was achieved by using duplex DNA to drive the connections of cells and thereby providing access to microtissues [21–22] or extracellular matrices [23–24] by DNA-based 2D-arrays.

DNA bears the inherent potential, that nature evolved a large toolbox of different enzymes for manipulation of DNA. These enzymes can be used to manipulate DNA for the construction of DNA nanometer scaled objects. For example, DNA ligases were applied to covalently attach DNA strands to each other to form covalently linked objects [16,18,25–26]. Furthermore, by using branched DNA constructs and ligases, a DNA hydrogel was generated [27–29]. Luo et al. used a DNA based network, manufactured in that fashion, that can act as a protein producing gel and can be used as an efficient cell-free translation system [30]. Recently, ordered 2D DNA scaffolds were reported in which a nanometer precise arrangement of enzymes on these scaffolds leads to efficient enzymatic communication [31]. DNA polymerases have been applied for the assembly of DNA nanostructures. Joyce et al. employed a DNA polymerase to synthesize long single-stranded DNA that folds into an octahedron by assistance of scaffolding DNA oligomers [17]. In another approach rolling circle amplification was used for enzymatic amplification of DNA nanostructures [32–33]. Recently, this approach was extended to operate in cells [34–35].

Furthermore, it has been shown that branched DNA constructs can form materials by self-assembly [36]. Richert et al. were able to generate DNA based materials based on branched DNA molecules which are non-covalently bound to each other by only hybridization of 2 nucleotides [37]. Interestingly, the formed DNA networks are remarkably stabilized (up to 95 °C) compared to the non-branched counterparts.

We previously reported an approach to construct three dimensional DNA networks that were generated and amplified by DNA polymerase chain reactions (PCR). In order to construct the network we developed covalently connected, 3-armed bDNA constructs (Y-motif) that act as primer and reverse primer strands in PCR [38–40]. The branching of the DNA was realized via a flexible alkyl chain that was connected to the nucleobase. Although the primer strands were covalently connected, they were accepted by a DNA polymerase and DNA networks formed by the enzyme. Based on this observation, we aim at investigating the impact of geometric constrains within the covalently branching unit on the network forming behavior of the branched DNA and the ability of DNA polymerases to form DNA networks by PCR. Thus, we developed a synthetic strategy for branched DNA by using a rigid branching point (Bp), based on the 1,3,5-triethynylbenzene scaffold. After the synthesis, the branched DNA was investigated towards its properties in network formation by self-assembly and PCR. We found that, despite the geometric restriction of the branching unit, the enzymatic generation of complex DNA networks by PCR was feasible. The novel generated DNA networks were investigated by agarose gel electrophoresis, atomic force microscopy, dynamics light scattering, and electron paramagnetic resonance spectroscopy on surfaces and in solution.

## Results and Discussion

**Scheme 1:**
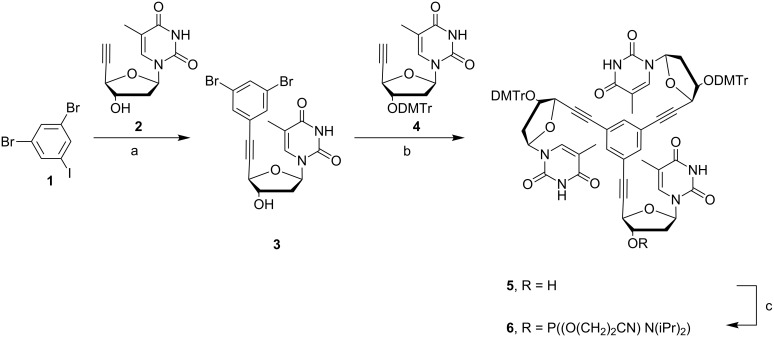
Reagents and conditions: (a) PdCl_2_(PPh_3_)_2_, DMF, CuI, NEt_3_, 55 °C, microwave, 82%; (b) PdCl_2_(PPh_3_)_2_, DMF, CuI, NEt_3_, 80 °C, microwave, 48%; (c) CH_2_Cl_2_, 2-cyanoethyl-*N*,*N*-diisopropylchlorophosphoramidite, *N*,*N*-diisopropylethylamine, 0 °C, 84%.

**Design and synthesis of the branching molecule**. In order to investigate the impact of rigidity of the branching core on DNA hybridization and usage of the constructs for network formation by PCR, we aimed at synthesizing a branching molecule based on the 1,3,5-triethynylbenzene scaffold. The oligonucleotides should be directly fused to the benzene ring via the three acetylene modifications resulting in a Y-shape topology. Thereby a rigid core with reduced degrees of rotation will be generated in contrast to other approaches that used more flexible branching molecules. The synthesis strategy was designed to meet the requirements of standard DNA solid support synthesis. Stepwise Sonogashira reaction was employed using the higher reactivity of iodide in the presence of bromide within 1,3-dibromo-5-iodobenzene (**1**) employing the known compounds **2** [41] and **4** [42] yielding **5** in acceptable yields (Scheme 1). Finally, compound **5** was transformed into **6** by phosphitylation resulting in a building block that bears protection groups and reactive groups that are standard in solid phase DNA oligonucleotide synthesis.

**Scheme 2:**
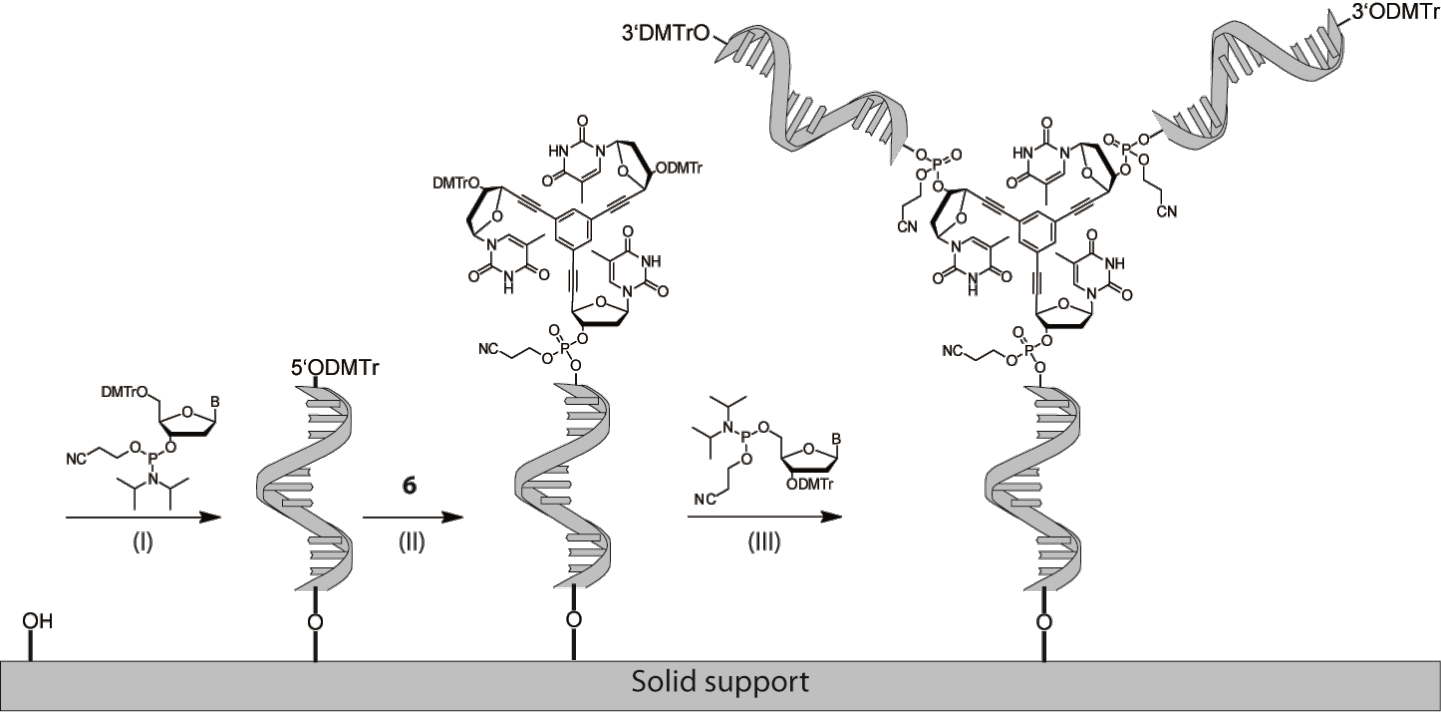
Stepwise solid-phase synthesis for branched oligonucleotides. (I): The first oligonucleotide branch is synthesized in 3'–5' direction using standard phosphoramidites. (II): Incorporation of the branching point by usage of **6**. (III): Simultaneous synthesis of the two remaining oligonucleotide branches in 5'–3' direction using inverse protected phosphoramidites.

**Synthesis of branched oligonucleotides.** DNA oligonucleotide synthesis was performed at 0.2 µmol scale (trityl-on mode) employing the standard phosphoramidites and **6** which was diluted in a mixture containing 10% CH_2_Cl_2_ in CH_3_CN to a final concentration of 0.12 M. 3000 Å LCAA-CPG support was used, derivatized with the respective 3'-nucleotide of the respective DNA oligomers. Since we later intended to investigate whether the oligonucleotide branches are used as primers in PCR (vide infra), the oligonucleotides have to terminate with a free 3'-hydroxy group. This requires a particular synthesis strategy (Scheme 2).

**Figure 1:**
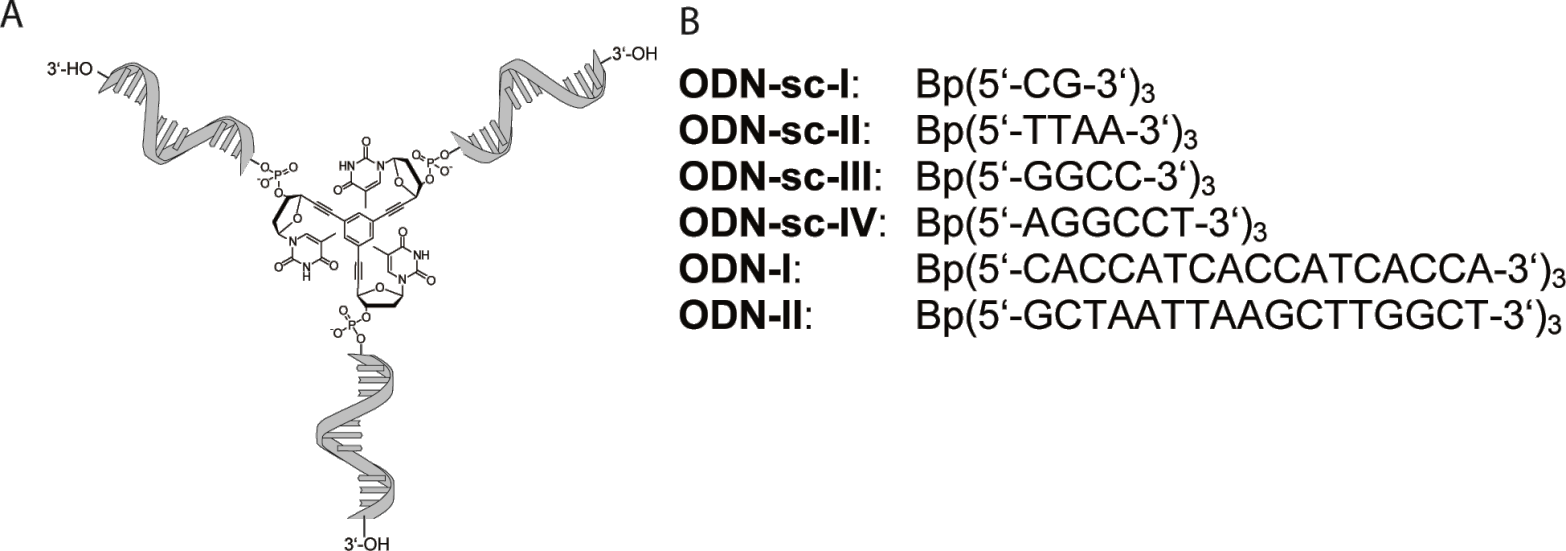
(A) Depiction of synthesized branched oligonucleotides; (B) Sequences of all synthesized branched oligonucleotides. Bp: branchpoint.

The synthesis strategy was adapted in a way that all branches have the same sequence and terminate with a free 3'-OH group required for processing by DNA polymerases. This was achieved by the employment of standard phosphoramidites until the incorporation of the branching unit **6**. Afterwards, the inverse-phosphoramidite strategy was used for the synthesis of both DNA strands. Following this approach a series of branched oligonucleotides were synthesized (Figure 1). The average coupling yield was always higher than 95% requiring a coupling time of 5 min only for the reaction of **6**. DNA-oligomers were purified twice by HPLC and characterized by ESI–IT–MS (see Supporting Information File 1).

**Characterization of bDNA by thermal denaturation studies and CD spectroscopy.** Formation of stable duplexes with complementary DNA strands is a prerequisite for the employment of the bDNA **ODN I** and **ODN II** in PCR experiments. Thus, we investigated duplex formation properties. In order to investigate whether the thymidine residues that are directly linked to the benzene core are amenable to participate in duplex formation, complementary oligonucleotides with varied lengths were investigated and compared to linear, non-branched reference duplexes. The thermal denaturation studies (see Supporting Information File 1) indicate that dependent on length, Y-shaped bDNA **ODN I** behave comparably to the linear non-branched counterpart **7** (Table 1). Increases in *T*_m_ were observed with increasing duplex length. Noteworthy, the obtained results show that the terminal thymidine that is covalently connected to the benzene core via a rigid ethylene bridge is amenable to contribute to duplex stability. This was evidenced by an increase in *T*_m,_ when **ODN I** was hybridized to **10** in comparison to the one nucleotide shorter **9**.

**Table 1:** Thermal denaturation studies comparing linear and branched oligonucleotides hybridization. Incorporated phosphoramidite **6** is depicted as Bp for branched **ODN I**.^a^

Linear DNA			bDNA		
	
duplex		*T*_m_ [°C]	duplex		*T*_m_ [°C]
					
5'-TGGTGATGGTGATGGT 3'-ACCACTACCACTACCACT	**8 7**	61.5	5'-TGGTGATGGTGATGGT (3'-ACCACTACCACTACCACT)_3_ Bp	**8 ODN I**	60.4
5'-TGGTGATGGTGATGGTG 3'-ACCACTACCACTACCACT	**9 7**	63.0	5'-TGGTGATGGTGATGGTG (3'-ACCACTACCACTACCACT)_3_ Bp	**9 ODN I**	62.4
5'-TGGTGATGGTGATGGTGA 3'-ACCACTACCACTACCACT	**10 7**	65.2	5'-TGGTGATGGTGATGGTGA (3'-ACCACTACCACTACCACT)_3_ Bp	**10 ODN I**	64.0
5'-TGGTGATGGTGATGGTGAC 3'-ACCACTACCACTACCACT	**11 7**	63.6	5'-TGGTGATGGTGATGGTGAC (3'-ACCACTACCACTACCACT)_3_ Bp	**11 ODN I**	62.3

^a^Temperatures were determined with ±0.5 °C accuracy.

Next, bDNA constructs were characterized by circular dichroism (CD) spectroscopy. The CD spectra of Y-shaped bDNA **ODN I** bound to oligonucleotides **8**–**11**, respectively, show dichroic peaks similar to those of unmodified DNA duplexes, indicating that bDNA maintain the B-DNA form (see Supporting Information File 1).

**Figure 2:**
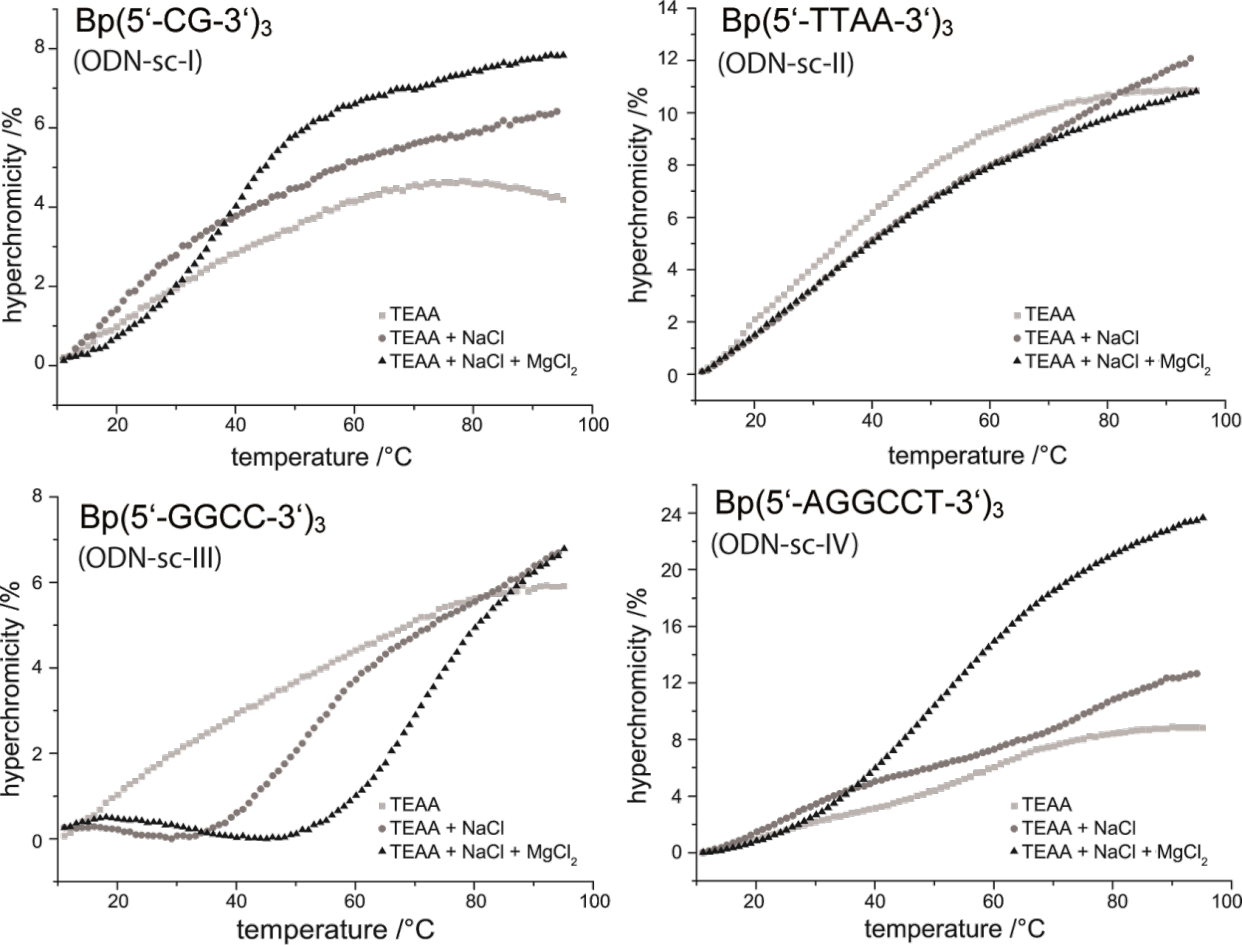
Thermal denaturating studies of self-complementary branched oligonucleotides. Different conditions are indicated in the figure. TEAA: triethylammonium acetate.

As the Y-shaped bDNA is able to hybridize with complementary linear strands, we next investigated, if these constructs can undergo self-assembly to form DNA networks. For this purpose, self-complementary (sc) bDNA constructs were synthesized (**ODN-sc-I** to **ODN-sc-IV**, cf. Figure 1) and the melting characteristics were addressed by thermal denaturation studies (cf. Figure 2).

The self-complementary oligonucleotides (5 μM) were annealed in 10 mM triethylammonium acetate buffer at pH 7 in the presence of sodium chloride (150 mM), sodium chloride and magnesium chloride (150 mM and 100 mM, respectively) or in the absence of salts. The solutions were heated and the UV absorbance was recorded in dependence on the temperature.

Interestingly, self-complementary constructs with high GC-content show higher melting temperatures and a strong increase of the melting temperature on the addition of Mg^2+^, whereas the linear control oligonucleotides showed no melting in all buffers tested (data not shown). In magnesium ion-containing buffer the CG 2-mer (**ODN-sc-I**) shows a higher melting temperature (37.6 °C) as the **ODN-sc-II** 4-mer (24.3 °C). The GC-rich 4-mer **ODN-sc-III** shows high melting temperatures even in the presence of only sodium chloride (58.0 °C) and an even higher melting temperature in the presence of additional magnesium (73.0 °C). The melting temperature in presence of sodium chloride and magnesium is even higher than the respective melting temperature of the 6-mer **ODN-sc-IV** (50.7 °C). All in all, one can conclude that the self-assembly of GC-rich self-complementary constructs is stronger than the self-assembly of AT containing constructs. Furthermore, the self-assembly is much stronger in the presence of magnesium ions for the GC-rich constructs.

**Figure 3:**
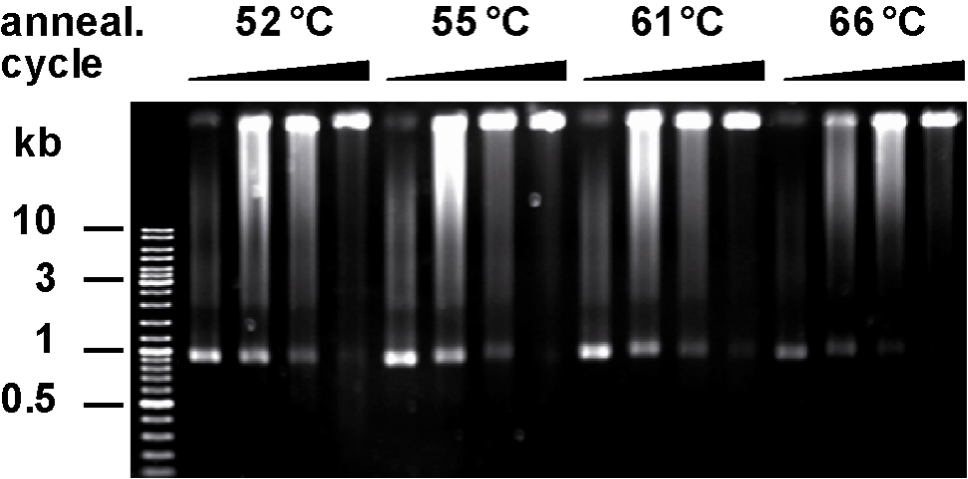
Generation of DNA networks with *Taq* DNA polymerase from 1062 nt template using primer **ODN I** and **ODN II**. Monitoring of DNA network growth influenced by varying of annealing temperature (as indicated) and increasing cycles (10, 18, 22, 28, respectively).

**Enzyme catalyzed network growth.** Next we investigated whether the synthesized **ODN I** and **II** are suitable primers for DNA network formation by PCR. We used a 1062 nt open reading frame of human DNA polymerase β as template and *Thermus aquaticus* (*Taq*) DNA polymerase for amplification. In earlier studies [17] of flexible Y-motifs strong dependence of the network formation on the annealing temperature was found. Therefore, we varied this parameter of PCR and investigated product formation by agarose gel electrophoresis (Figure 3).

Depending on the cycle number the formation of slower migrating products was observed. At the highest cycle number (28 cycles) at all investigated annealing temperatures ranging from 52–66 °C the formation of DNA networks with hardly any mobility was observed. Noteworthy, using linear primer strands of the same sequence resulted in the formation of the expected linear reaction products that migrated as expected (see Supporting Information File 1). No amplification products were observed in the absence of template DNA under identical conditions (see Supporting Information File 1).

**Characterization of DNA networks.** The formed DNA networks were studied by atomic force microscopy (AFM) using the tapping mode [43–44]. The original sample solutions were diluted to a total amount of 10 ng/µL DNA for AFM measurements with buffer (10 mM Tris, pH 7.4, 1 mM NiCl_2_). Freshly cleaved mica was incubated with the sample following a multistep protocol (see Supporting Information File 1).

**Figure 4:**
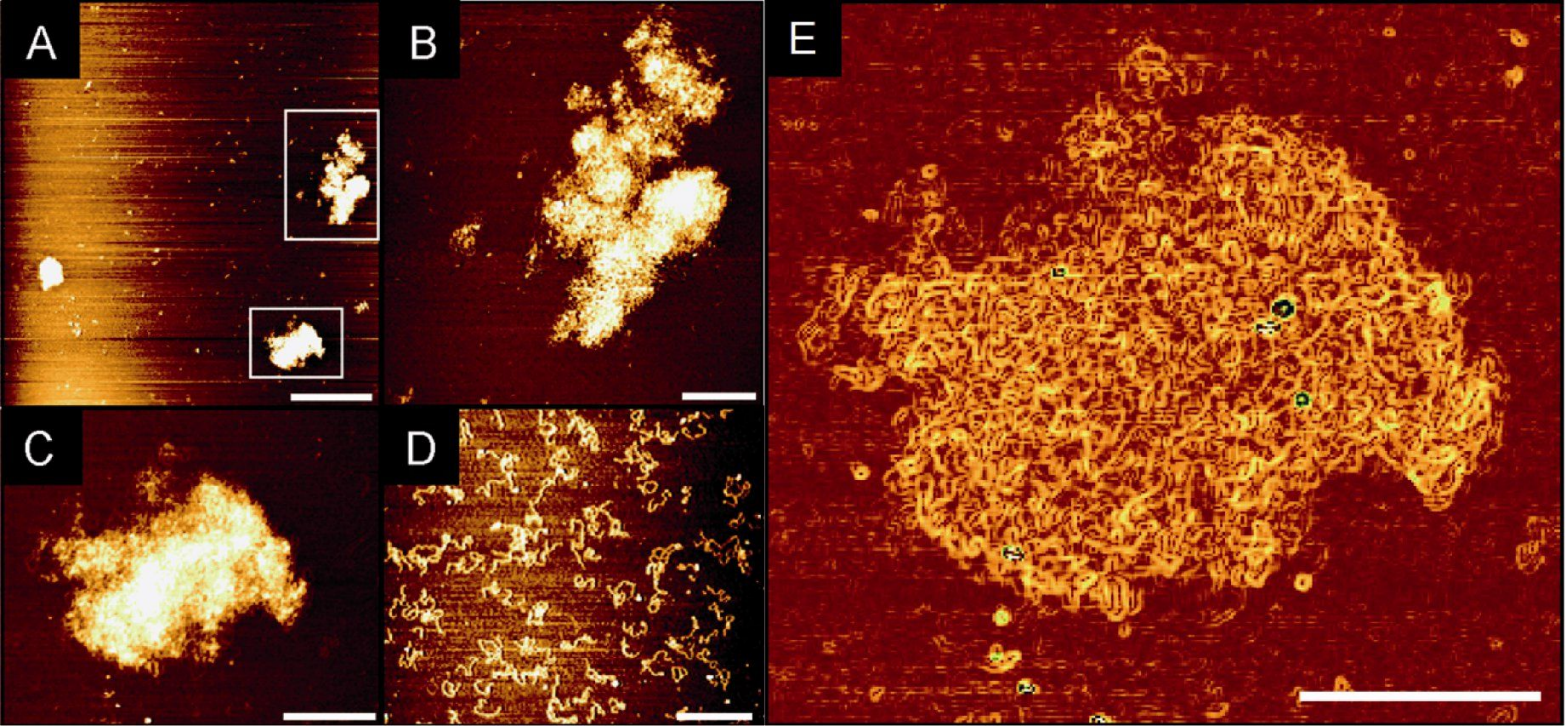
AFM analysis of DNA structures: (A) Overview-scan 10 × 10 µm showing DNA networks generated after 28 cycles with 66 °C annealing temperature (bar is 2 µm). (B, C) Zoom in picture of DNA network (all bars are 0.5 µm, respectively). (D) AFM when non-branched primers were employed (bar is 0.5 µm). (E) Software analysis of AFM measurement taking first derivative of height channel. DNA network generated after 28 cycles with 66 °C annealing temperature (bar is 0.5 µm).

As control, the PCR products of standard linear primer strands were investigated first (Figure 4D). Long linear double stranded DNA (dsDNA) is flexible. During AFM measurement linear or coiled structures with a height ranging from 0.5 to 1.1 nm (theoretic diameter 2 nm) and a width of about 15 nm were observed. Due to the force, which is applied by the scanning tip, the DNA is flattened, which might explain the smaller height of the observed DNA [45]. The increased width of the observed objects is a result of the finite size of the scanning tip, leading to a shape broadening of objects [46]. The PCR products derived from branched primer strands **ODN I** and **II** were found to be extended DNA networks (Figure 4A–C). An overview scan of 10 × 10 µm (Figure 4A) showed the diversity of shapes. Further AFM scans with higher resolution (Figure 4B,C) depicted DNA networks with dimensions from 0.6 to 2 µm in the surface dimension and a height up to 7 nm (see Supporting Information File 1). These findings correlate well with the decreased mobility of the structures in the agarose gel electrophoresis (Figure 3) demonstrating one covalently connected migrating DNA molecule. The irregular shapes of the DNA networks might result from the collapse of three-dimensional structures forced by ionic interactions and induced by nickel ions on the mica surface.

More information about the DNA network character was observed taking the first derivative of the height output channel resulting in Figure 4E. The principle of this mathematic operation is to assume a local maximum at an edge resulting from cross section measurements of objects (see Supporting Information File 1). Using this operation the data depicted in Figure 4C could be transferred into the data depicted in Figure 4E. The AFM picture showed a better contrast for the visualization of DNA networks. Proceeding in this way, the DNA strands were better resolved and gave an impression of the shape of DNA networks. The observed DNA networks showed a less ordered shape which can be related to DNA flexibility. This flexibility was also observed with linear DNA (Figure 4D).

In order to review whether DNA networks form in solution the network generated by PCR was investigated by dynamic light scattering (DLS). The obtained data indicate that DNA networks are also present in solution. The measured average hydrodynamic diameter (*D*_H_) at 90° after 10 measurements were partially 67 nm and 593 nm while the linear DNA showed a *D*_H_ of 11 nm (see Supporting Information File 1). This *D*_H_ is variable at different angles because DNA networks were not expected to have spherical morphology in solution [47]. Furthermore, different *D*_H_ values in the case of branched DNA samples were owed to the dynamics in solution. Comparing scattering intensity of linear DNA and branched DNA networks, gave the possibility to conclude that DNA networks existed in solution in variable shapes.

Next we employed electron paramagnetic resonance (EPR) spectroscopy for investigation of the DNA networks. EPR is a widespread technique for the studies of structural and dynamic properties of biological macromolecules, e.g., DNA [48–53]. Since the systems studied in the current investigation are diamagnetic, nitroxide labels had to be inserted enzymatically [54–55]. EPR spectra of nitroxide labels are sensitive to dynamics on the picosecond to microsecond timescales and these dynamics are greatly altered upon attachment to a macromolecule. The nitroxide labels report not only the dynamics of the macromolecules as a whole, but additionally, they undergo rotations around the chemical bonds of the linker, and furthermore, the site of attachment can undergo conformational changes compared to the rest of the macromolecule. As the EPR-signal arises from these three processes, the interpretation of EPR spectra is rendered difficult. Measurements at a single frequency do not allow complete description of the spin label motion. However, a spectrum often can be approximated by a simple motional model to provide information on the properties of the macromolecule [56].

Since DNA polymerases are known to tolerate several dNTP modifications [57–58], we next investigated whether dTTP can be partially replaced by the nitroxide labeled dT*TP to generate DNA networks containing spin labels covalently bound to DNA.

**Figure 5:**
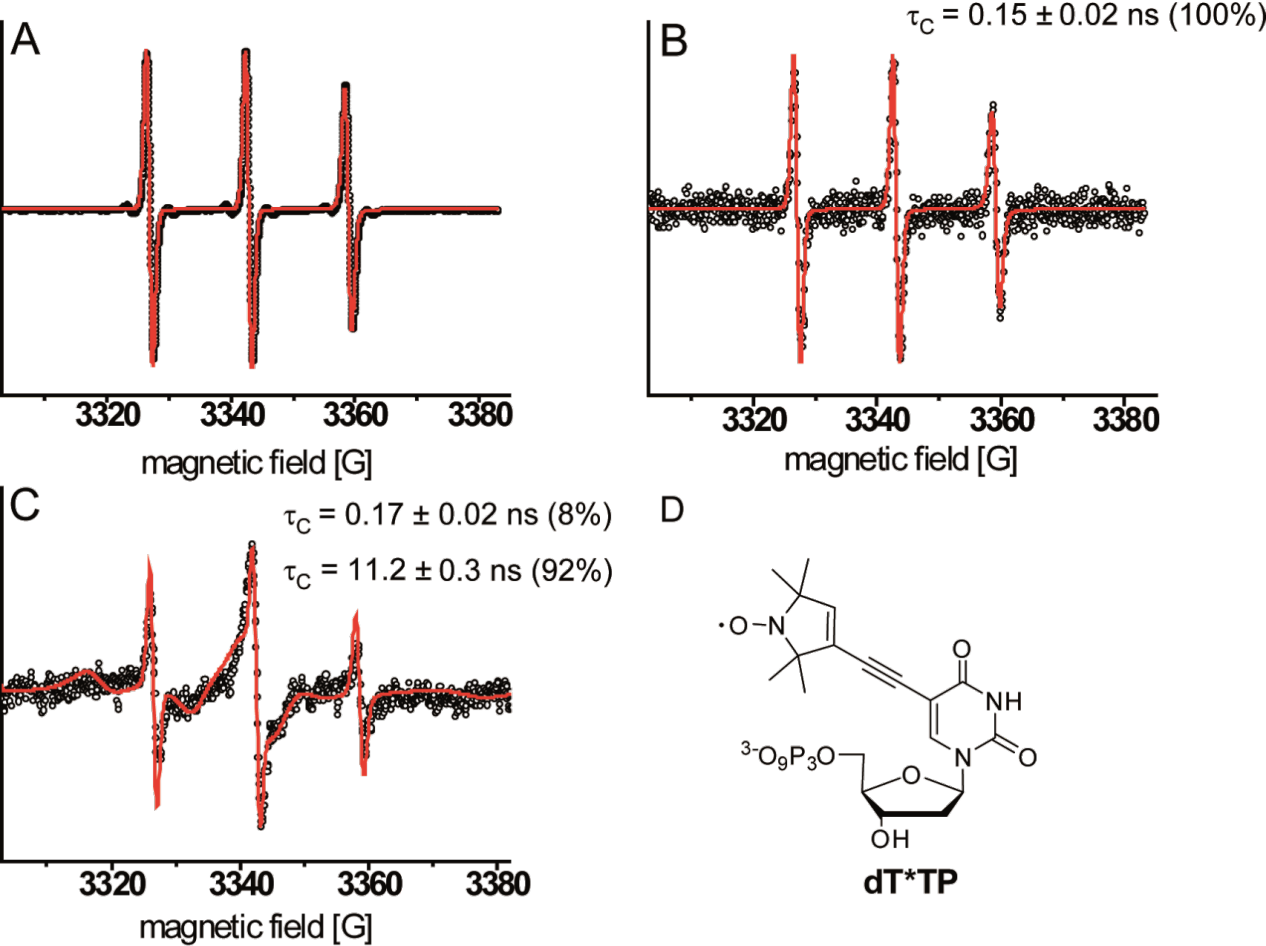
EPR spectra with corresponding spectral simulations (red line) of (A) dT*TP, (B) PCR reaction product resulting from non-branched primers in the presence of 50% dT*TP; The simulated spectrum and the corresponding τ_C_ are derived from a one component fit, (C) DNA networks generated by PCR in the presence of 50% dTTP* using branched primer; the simulated spectrum and corresponding τ_C_ are derived from a two component fit, (D) structure of modified dT*TP employed in PCR.

All EPR spectra were measured in X-band (9.5 GHz) at room temperature in solution and are shown as first derivatives. For later comparison, spin-labeled TTP analogue dT*TP (Figure 5D) was measured and results to EPR spectra with three narrow lines indicating a high rotational mobility averaging the anisotropy of the hyperfine interaction to the ^14^N nucleus (Figure 5A) [54].

Using the same approach as described above, we generated a linear DNA construct as well as a DNA network using a 1062 bp long template in the presence of a 1:1 ratio of dT*TP to TTP and natural dNTPs. We obtained spectra showing that the spin-labeled nucleotide was indeed incorporated into the DNA (Figure 5B,C). All spectra were quantitatively analyzed by spectral line shape simulations. Thereby, the dynamics of the nitroxide spin-labels are reflected in rotational correlation times τ_c_ assuming isotropic rotation of the label. The ^13^C-satellites (Figure 5A) were not taken into account for simulations. While spectra of Figure 5A and B are satisfactorily described by this approach, two components featuring two different rotational correlation times τ_c_ were required in case of Figure 5C. The two component fit was more consistent than assuming anisotropic rotational diffusion or a log-Gaussian distribution of correlation times [59]. The main component of the spectrum features a drastically reduced rotational mobility of the spin-label. The rotational correlation times as well as the fraction of both spectral components for spin labeled DNA networks as derived by spectral simulations are summarized in Table S1 (see Supporting Information File 1). The drastic decrease in mobility of the probe shown in the EPR spectrum upon enzyme-catalyzed DNA network growth (Figure 5C) clearly indicated network formation in solution and suggests rather rigid DNA networks. A more detailed analysis of the spectrum results in two components; the first component (contributing 8%) features a rotational correlation time (τ_c_ = 0.17 ± 0.02 ns) almost identical to τ_c_ of spin labels in linear DNA (τ_c_ = 0.15 ± 0.02 ns). The second component (92%) gives rise to a correlation time of τ_c_ = 11.2 ± 0.3 ns and is allocated to spin-labels incorporated into the DNA network. Since the presence of linear, non-branched DNA in the sample was excluded by gel filtration, we concluded, that the first component originated from “dangling DNA” at the edges of DNA networks. In a simple model, two-dimensional, approximately circularly shaped networks consisting of some 600 hexagons would contain about 8% dangling DNA. The diameter of such a structure could be estimated to several micrometers and is thus consistent with the AFM results. Concentrations of spin labels were obtained from the simulations fitted to the experimental data and used to derive the degree of labeling. We found that 1% of incorporated thymidines were replaced by the spin labeled analogue in PCR when linear strands as well as networks were formed. Thus, one dsDNA strand contains approximately 4 incorporated spin labels.

## Conclusion

To summarize, branched DNA primers were applied for the generation of DNA networks via enzymatic elongation of DNA by PCR. A straightforward synthesis was developed resulting in a rigid DNA branching building block **6** that was successfully used in solid phase DNA synthesis. In thermal denaturation studies Y-shaped bDNA indicates comparable behavior to the linear non-branched counterpart, thus forming a B-DNA conformation. Furthermore, the branched DNA self assembles into stable networks when short self-complimentary DNA sequences are used. We found that despite its rigidity, *Taq* DNA polymerase accepts the branched DNA construct as primers and builds DNA networks that grow cycle by cycle in PCR. Further, the generated DNA networks can be visualized using AFM. Generated images present surface covering structures in different shapes which are further characterized in solution by DLS. EPR measurements further corroborate network formation and suggest rather rigid DNA networks. As demonstrated, the approach allows to additionally modify the networks by using chemically modified nucleotides during PCR. The depicted approach might find applications like in the generation of functional hydrogels or tissue engineering.

## Supporting Information

Experimental procedures and full characterization data for all new compounds, materials and general procedures are given. Oligonucleotide synthesis and characterization of oligonucleotides, original melting curves of oligonucleotides and CD spectra of double stranded Y-motif are also shown. Further AFM studies, DLS spectra and EPR related experiments.

File 1 Experimental part.
