# Supplementary material for: A new building block for DNA network formation by self-assembly and polymerase chain reaction
Source: Beilstein J Org Chem. 2014 May 7;10:1037–46. doi: 10.3762/bjoc.10.104 (PMC4077517; doi:10.3762/bjoc.10.104)
Supplement: File 1 — Experimental part. [file Beilstein_J_Org_Chem-10-1037-s001.pdf]

Supporting Information

for

A new building block for DNA network formation by self-assembly and polymerase chain reaction

Holger Bußkamp[‡], Sascha Keller[‡], Marta Robotta, Malte Drescher and Andreas Marx*[§]

Address: Department of Chemistry and Konstanz Research School Chemical Biology, University of Konstanz, Universitätsstraße 10, 78457 Konstanz, Germany

Email: Andreas Marx - Andreas.Marx@uni-konstanz.de

*Corresponding author

[§]Tel. +49-7531-88 5139; Fax. +49- 7531-88 5140;

[‡]contributed equally

Experimental part

Table of contents

1) General S2

2) Synthesis DNA branching building block. S3

3) Synthesis of oligonucleotides S6

3) Characterization of duplex formation S7

4) Determination of melting characteristics of self complementary oligonucleotides S9

5) Characterization of DNA networks S9

1) General. Chemicals for synthesis of the DNA-branching building blocks were purchased from Acros, Aldrich, Sigma, or Fluka (Sigma-Aldrich, Germany). Solvents were of laboratory grade. Thin-layer chromatography (TLC) was performed on TLC aluminium sheets covered with silica gel 60 F254 (0.2 mm, Merck, Germany). Column flash chromatography (FC): silica gel 60 (Merck, Germany) at 0.3 bar. Microwave assisted reactions were performed on an Initiator EXP EU (Biotage, Sweden). ESI–IT mass spectra were recorded on a Bruker Daltonics esquire 3000plus. High-Resolution ESI–TOF mass spectra were recorded by using a micrOTOF II (Bruker Daltonics). NMR spectra were recorded at 298 K using Avance 400 (^1H = 400 MHz, Bruker, Germany) and on an Avance 600 (^1H = 600 MHz, Bruker, Germany). *J* values are given in Hz; δ values in ppm relative to Me_4Si as internal standard, or 85% H_3PO_4 for ^{31}P. DNA oligonucleotides were synthesized on an Applied-Biosystem 392 DNA/RNA synthesizer or purchased from Ella Biotech (Germany). Chemicals and solid support for DNA synthesis were purchased from AzcoBiotech (USA), J.T. Baker (Netherlands) and Link-Technologies (Scotland, UK). Reversed-phase HPLC was performed using a Prominence HPLC (Shimadzu, Japan) instrument equipped with a Nucleosil-100-5 C18 column (250 × 4 mm, Macharey-Nagel, Germany). Thermal denaturation studies were performed using a Cary-100 Bio UV–vis spectrophotometer (Varian, Australia) equipped with a Cary Thermo-controller using a heating rate of 1 $°C \cdot min^{-1}$. CD spectra were recorded on a Jasco 715 instrument (Jasco Inc., Easton Maryland). DLS measurements were performed using a Nano ZS (Malvern Instruments) instrument. PCR was performed using a PCR-Thermocycler system (BIOMETRA) and dNTPs were purchased from Fermentas.

QIAquick Gel Extraction Kit and MinElute Reaction Cleanup Kit were from Qiagen. Agarose-gels were photographed using ChemiDoc XRS from BioRad. AFM images were acquired on a NanoWizard II (JPK Instruments, Germany) using high-grade Mica (1 × 1'', TED Pella, USA) and cantilevers from APPNano (CA, USA) and Nanoworld (Switzerland).

2) Synthesis DNA branching building block.

1-((2*R*,4*S*,5*R*)-5-((3,5-dibromophenyl)ethynyl)-4-hydroxytetrahydrofuran-2-yl)-5-thymidine (3)

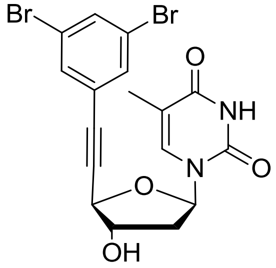

In an inert atmosphere, NEt_3 (2 mL) was added to a stirred solution of **2** (0.43 g, 1.81 mmol), 1-iodo-3,5-dibromobenzene (**1**, 0.69 g, 1.90 mmol), $(PPh_3)_2PdCl_2$ (24.4 mg, 0.036 mmol) and CuI (13.8 mg, 0.072 mmol) in DMF (2 mL). The resulting solution was heated with a microwave (max. 350 W) for 15 min at 55 °C. The solvent was removed in vacuo and the remaining solid was purified by silica-gel column chromatography (1–5% MeOH/CH_2Cl_2) to yield **3** as a yellowish solid (0.68 g, 80%). R_f 0.51 (CH_2Cl_2 /MeOH 10:1); ^{1}H NMR (400 MHz, DMSO-d_6) δ 11.38 (s, 1H, N-H), 7.93 (t, J = 1.7 Hz, 1H, p-arom.), 7.78 (d, J = 1.7 Hz, 2H, o-arom.), 7.56 (s, 1H, H_6), 6.34 (dd, J = 7.8, 6.6 Hz, 1H, $H_{1'}$), 4.74 (s, 1H, $H_{4'}$), 4.51 (s, 1H, $H_{3'}$), 2.37 – 1.97 (m, 2H, $H_{2'}$), 1.70 (s, 3H, $CH_{3,thymidine}$).^{13}C NMR (101 MHz, DMSO-d_6) δ 163.60, 150.56, 135.97, 134.3, 133.06, 125.05, 122.56, 109.59, 89.60, 85.42, 84.41, 76.21, 75.74, 38.17, 12.23. MS (ESI$^+$) m/z (calcd) 470.9 ($[M+H]^+$, 100%), m/z (found) 470.5; HRMS-ESI$^+$ m/z (calcd for $C_{17}H_{14}Br_2N_2O_4$ $[M+H]^+$) 470.9374, m/z (found) 470.9352.

Compound 5

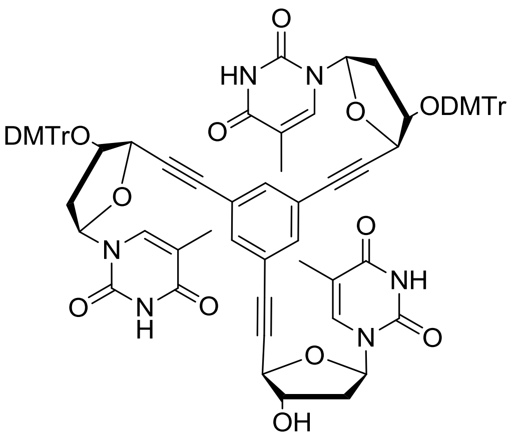

In an inert atmosphere, NEt_3 (1.6 mL) was added to a stirred solution of **4** (0.64 g, 1.20 mmol), **3** (0.27 g, 0.58 mmol), $(PPh_3)_2PdCl_2$ (8.1 mg, 0.012 mmol), and CuI (2.2 mg, 0.012 mmol), in DMF (3 mL). The resulting solution was heated within a microwave (max. 350 W) for 80 min at 80 °C. The solvent was removed in vacuo and the remaining solid was purified by silica-gel column chromatography (1–5% MeOH/CH_2Cl_2) to yield **5** (0.35 mg, 44%) after recrystallization in hexane as yellowish powder. R_f 0.26 (CH_2Cl_2/MeOH 95:5). For NMR signal nomenclature 3′-OH moiety containing signals are called A and 3′-DMTr moiety containing signals are called B. ^{1}H NMR (400 MHz, acetone-d_6) δ 7.59 – 7.20 (m, 24H, overlapping signals $H_{6\ A,B}$, $H_{arom.\ core}$, H_{DMTr}), 6.88 (dd, J = 9.0, 3.1 Hz, 8H, $H_{ortho\ to\ MeO}$), 6.53 – 6.44 (overlapping signal: 6.49 (dd, J = 8.1, 6.4 Hz, 2H, $H_{1'A}$); 6.47(dd, J = 7.9, 6.3 Hz, 1H, $H_{1'B}$)), 4.89 (s, 1H, $H_{4'A}$), 4.66 (d, J = 4.5 Hz, 1H, $H_{3'A}$), 4.54 (d, J = 5.1 Hz, 2H, $H_{3'B}$), 4.12 (s, 2H, $H_{4'B}$), 3.78 (s, 6H, H_{MeO}), 3.77 (s, 6H, H_{MeO}), 2.52 – 2.32 (m, 4H, $H_{2'B}$), 2.25 – 2.15 (m, 2H, $H_{2'A}$), 1.70 (s, 3H, $CH_{3\ thymidine,\ A}$), 1.68 (s, 6H, $CH_{3\ thymidine,\ B}$). ^{13}C NMR (101 MHz, acetone-d_6) δ 164.41, 159.94, 151.39, 145.92, 136.63, 136.59, 136.55, 136.38, 135.37, 135.27, 131.14, 131.11, 129.04, 128.95, 128.02, 124.08, 124.01, 114.35, 114.33, 111.14, 111.12, 88.91, 88.63, 87.43, 87.30, 86.35, 80.25, 77.90, 77.38, 75.93, 40.25, 39.28, 13.14, 13.07. MS (ESI$^-$) m/z (calcd) 1419.5 ([M+Cl$^-$]$^-$, 100%), m/z (found) 1419.4; HRMS-ESI$^-$ m/z (calcd for $C_{81}H_{72}N_6O_{16}$ [M-H]$^-$) 1383.4921, m/z (found) 1383.4920.

Compound 6

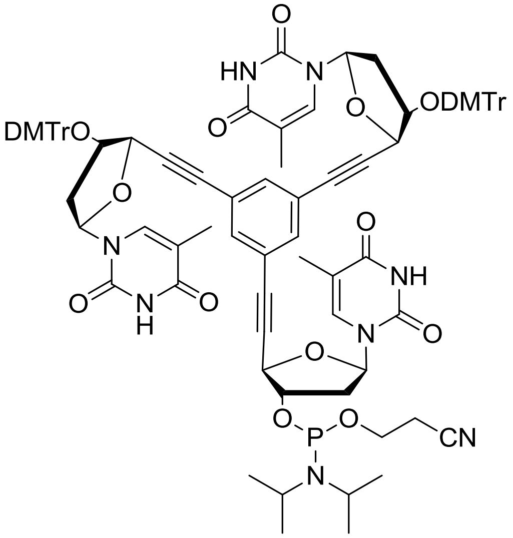

Compound **5** (244 mg, 0.18 mmol) was dissolved in dry CH_2Cl_2 and reacted with *N*-diisopropyl-2-cyanoethyl-chlorophosphoramidite (87 μL, 0.36 mmol) in the presence of diisopropylethylamine (245 μL, 1.4 mmol) at room temperature. The reaction mixture was stirred for 6 h under argon atmosphere. The mixture was poured into saturated Na_2CO_3 solution and washed with water (2×) and brine (2×). The organic layers were combined and dried over $MgSO_4$. After evaporation, the product was recrystallized from hexane to give **6** (240 mg, 84%) as white needles. R_f 0.37 (CH_2Cl_2 /MeOH 95:5); MS (ESI$^+$) m/z (calcd) 1585.6 ([M+H]$^+$, 100%), m/z (found) 1585.3; ^{31}P NMR (162 MHz, acetone-*d*$_6$) δ 149.76, 149.31. HRMS-ESI$^+$ m/z (calcd for $C_{90}H_{89}N_8O_{17}$ [M+Na$^+$]$^+$) 1607.5976, m/z (found) 1607.5910.

3) Synthesis of oligonucleotides

The DNA oligonucleotide synthesis was performed on an ABI 392-DNA/RNA-synthesizer (Applied Biosystem) at a 0.2 μmol scale (trityl-on mode) employing the standard phosphoramidites and building block **6** which was diluted in 10% CH_2Cl_2 in CH_3CN to a final concentration of 0.12 M. The synthesis was performed 3000 Å LCAA-CPG support. The average coupling yields were always higher than 95% by using a coupling time of 5 min for reaction with **6**. After cleavage from the solid-support, the oligonucleotides were deprotected in 33% aq NH_3 for 16 h at 60 °C. The DMT-containing oligonucleotides were purified by reversed-phase HPLC (RP-18) with the following solvent gradient system [A: 0.1 M (Et_3NH)OAc (pH 7.0)/CH_3CN 95:5; B: CH_3CN; gradient *I*: 0–3 min 10–15% B in A, 3–15 min 15–50% B in A, 15–20 min 50–10% B in A, flow rate 2 $mL{\cdot}min^{-1}$]. Then, the mixture was evaporated to dryness, and the residue was treated with 80% acetic acid for 30 min at 25 °C to remove the 4,4'-dimethoxytrityl residues. The detritylated oligomers were purified by reversed-phase HPLC with the gradient *II*: 0–20 min 5–20% B in A, 20–25 min 70% B in A, 25–30 min 70–5% B in A, flow rate 2 $mL{\cdot}min^{-1}$. The oligonucleotides were lyophilized on a Speed Vac evaporator to yield colourless solids which were stored frozen at −20°C.

ESI-MS analysis of synthesized branched oligonucleotides:

ODN-sc-I:	*m*/*z* (calcd.) 2634.5	*m*/*z* (found): 2634.5
ODN-sc-II:	*m*/*z* (calcd.) 4482.9	*m*/*z* (found): 4482.9
ODN-sc-III:	*m*/*z* (calcd.) 4488.8	*m*/*z* (found): 4488.8
ODN-sc-IV:	*m*/*z* (calcd.) 6340.1	*m*/*z* (found): 6340.1
ODN I:	*m*/*z* (calcd.) 16047.4	*m*/*z* (found): 16046.4
ODN II:	*m*/*z* (calcd.) 16569.5	*m*/*z* (found): 16569.3

3) Characterization of duplex formation

Thermal denaturation curves were measured with a Cary-100 Bio UV–vis spectrophotometer (Varian, Australia) equipped with a Cary thermo-controller using a heating rate of 1 °C·min^{-1} in a buffer containing KH_2PO_4 (20 mM, pH 7.0) and NaCl (200 mM) at room temperature. The samples contained 2 µM duplex DNA.

Corresponding to **Table 1**: Thermal denaturation spectra showing branched oligomer duplexes A: **8** + **ODN I**; B: **9** + **ODN I**; C: **10** + **ODN I**; D: **11** + **ODN I**.

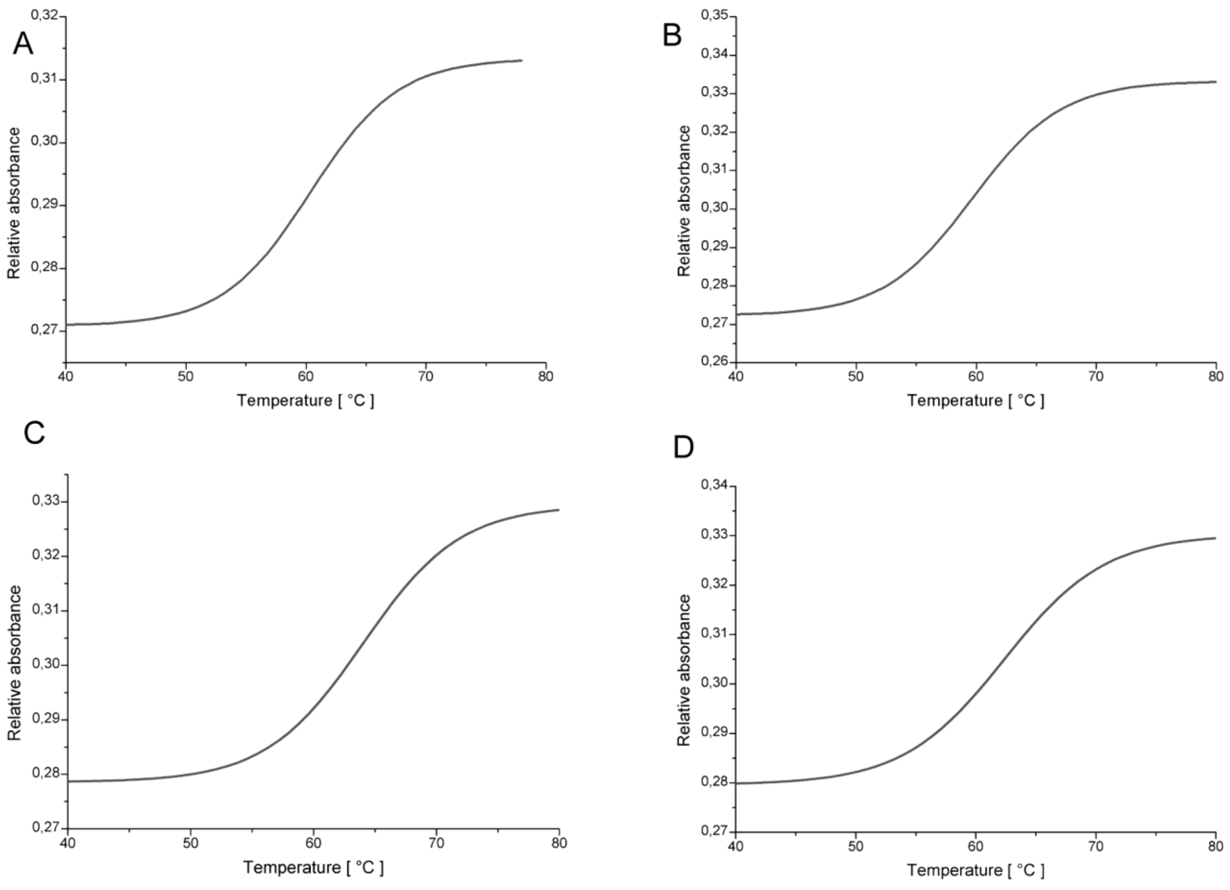

Figure S1: Thermal denaturation curves of DNA duplexes A, B, C, D, respectively.

USED OLIGOnucleotides:

7 5´-TCACCATCACCATCACCA

8 5´-TGGTGATGGTGATGGT

9 5´-TGGTGATGGTGATGGTG

10 5´-TGGTGATGGTGATGGTGA

11 5´-TGGTGATGGTGATGGTGAC

Corresponding CD spectra to thermal denaturation experiments A – D

Circular dichroism (CD) spectra were recorded on a Jasco 715 instrument (Jasco Inc., Easton Maryland) in a buffer containing KH_2PO_4 (20 mM, pH 7.0) and NaCl (200 mM) at room temperature. The samples contained 2 μM duplex DNA for the linear DNA motives. In the case of branched DNA motives linear DNA oligomers were used 6 μM and branched DNA motives 2 μM to saturate all three arms of branching DNA motive. All mixtures were heated to 95 °C for 5 min and allowed to cool slowly to room temperature prior to measurements. A spectrum of the buffer was measured separately and subtracted from the spectra resulting from the samples. An average of 10 spectra was recorded in each experiment.

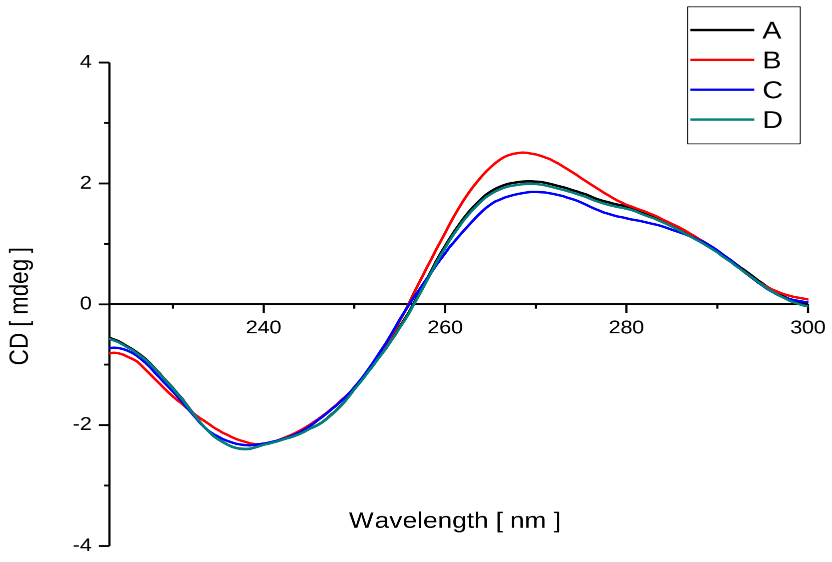

Figure S2: CD-spectra of DNA duplexes A, B, C, D, respectively.

4) Determination of melting characteristics of self complementary oligonucleotides

5 µM self-complementary oligonucleotides were dissolved in either 0.1 M triethylammonium acetate buffer (pH 7), or 0.1 M triethylammonium acetate buffer (pH 7), 150 mM NaCl or 0.1 M triethylammonium acetate buffer (pH 7), 150 mM NaCl and 100 mM $MgCl_2$. The corresponding linear controls were dissolved in the same buffers at a final concentration of 15 µM. The thermal denaturation curves were determined as described above.

5) Characterization of DNA networks

PCR was performed using a PCR-Thermocycler system (BIOMETRA). The reactions were performed containing 4 nM of the respective templates, 0.5 U of *Taq* DNA polymerase (Fermentas), 1× *Taq*-reaction buffer (75 mM TrisHCl (pH 8.8), 20 mM KCl and 0.01% Tween), 3 mM $MgCl_2$ and water were mixed in an overall volume of 20 µL.

The final mixtures contained dNTPs (200 µM each of dATP, dGTP, dCTP, and TTP), primers (0.5 µM each of the respective primer probe and reverse primer), 0.5 U *Taq* DNA polymerase (Fermentas; units defined by the supplier). PCR amplifications were performed by employing the following program: Initial denaturation at 95 °C for 3 min, followed by cycles of denaturation at 95 °C for 30 s, primer annealing at varying temperatures for 90 s, and extension at 72 °C for 60 s.

1062nt template sequence used in PCR:

pGDR11: pol Beta encoding Sequence ORF

```
5´-
CACCATCACCATCACCATACGGATCCGATGAGCAAACGTAAAGCGCCGCAGGAAACCCTGAACGGCGGC
ATTACCGATATGCTGACCGAACTGGCCAACTTTGAAAAAAACGTGAGCCAGGCGATCCATAAATATAAC
GCGTATCGTAAAGCGGCGAGCGTGATTGCGAAATATCCGCACAAAATTAAAAGCGGTGCGGAAGCGAAA
AAACTGCCGGGCGTGGGCACCAAAATTGCGGAAAAAATCGATGAATTTCTGGCCACCGGCAAACTGCGT
AAACTGGAAAAAATTCGCCAGGATGATACCAGCAGCAGCATTAACTTTCTGACCCGTGTGAGCGGCATT
GGTCCGAGCGCGGCGCGTAAATTTGTGGATGAAGGCATCAAAACCCTGGAGGATCTGCGTAAAAACGAA
GATAAACTGAACCATCATCAGCGTATTGGCCTGAAATATTTTGGCGATTTCGAAAAACGTATTCCGCGT
GAAGAAATGCTGCAGATGCAGGATATTGTGCTGAACGAAGTGAAAAAAGTGGATAGCGAATATATTGCG
ACCGTGTGCGGCAGCTTTCGTCGTGGCGCGGAAAGCAGCGGCGATATGGATGTGCTGCTGACCCATCCG
AGCTTTACCAGCGAAAGCACCAAACAGCCGAAACTGCTGCATCAGGTGGTGGAACAGCTGCAGAAAGTG
CATTTTATTACCGATACCCTGAGCAAAGGCGAAACCAAATTTATGGGCGTGTGCCAGCTGCCGAGCAAA
AACGATGAAAAAGAATATCCGCATCGCCGTATTGATATTCGTCTGATCCCGAAAGATCAGTATTATTGC
GGCGTGCTGTATTTTACCGGCAGCGATATCTTCAACAAAAACATGCGTGCGCATGCGCTGGAAAAAGGC
```

TTTACCATCAACGAATACACCATTCGTCCGCTGGGCGTGACCGGTGTTGCGGGTGAACCGCTGCCGGTG
GATAGCGAAAAAGATATCTTCGATTACATCCAGTGGAAATATCGTGAACCGAAAGATCGTAGCGAATAA
GTCGACCTGCAGCCAAGCTTAATTAGC-3´

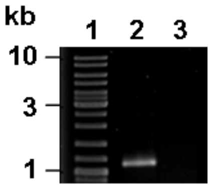

Figure S3: Agarose gel electrophoresis analysis of PCR. Lane 1: DNA ladder, Lane 2 PCR with non-branched primers, annealing temperature of 55 °C, 28 cycles; Lane 3: PCR with branched primers without template, annealing temperature of 55 °C, 28 cycles.

AFM:

The original sample solutions were diluted to 10 ng·μL^{-1} for AFM measurements with buffer (10 mM Tris pH 7.4, 1 mM $NiCl_2$). Freshly cleaved Mica (1 × 1'', TED Pella, USA) was incubated with 50 μL of the same buffer for 5 min, washed with 200 μL de-ionized water and dried under steam of nitrogen. 20 μL of the sample containing solution was dropped on Mica and handled after 5 min incubation time in the mentioned way. The mica sheet was then mounted in the AFM. Images were recorded in air using a commercial AFM (NanoWizard II (JPK Instruments, Germany)). Silicon cantilevers (ACTA-Probe, APPNano, California, 125 μm long, 35 μm wide, 4.5 μm thick) with a pyramidal shaped tip (spring constant of 40 Nm^{-1} and a resonance frequency of 300 kHz in air) were used.

In air, we selected a driving frequency around 300 kHz for imaging. AFM images (512 × 512 pixels) were recorded at a scan rate of 1 Hz. Images were processed by flattening to remove the background slope. Thereby JPK SPM Image Processing Software Version 3.3.25 was used.

Cross section of DNA networks

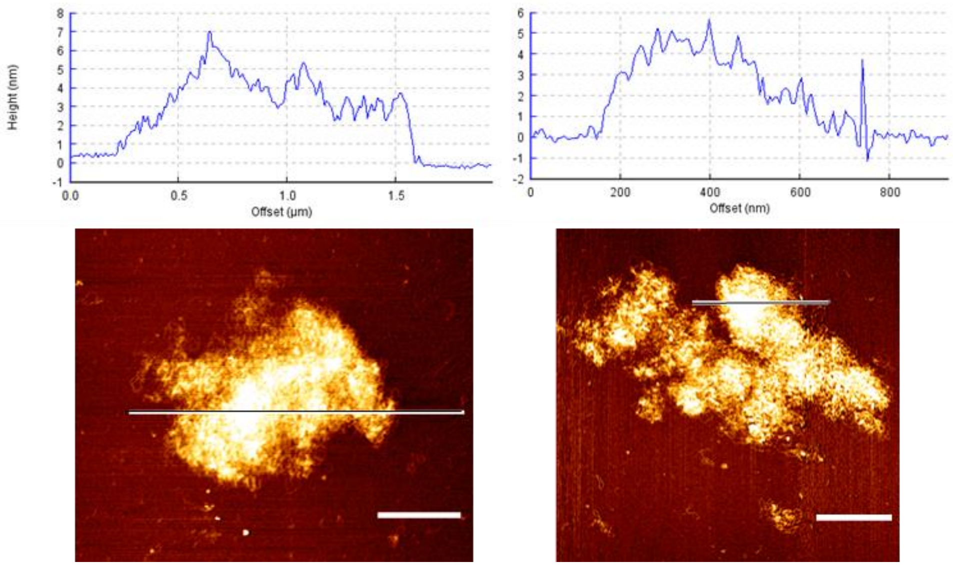

Figure S4: Cross section of two DNA networks, bar is 0.5 µm.

Dynamic Light Scattering (DLS) measurements were performed on a commercially available Nano ZS (Malvern Instruments) using PCR product of DNA containing samples. Note: The same PCR sample was prepared and mounted on the AFM.

A) Linear: particle size 10.8 nm, polarization 90°, 10 measurements in 60 sec at 25 °C, 100 µL PCR mixture

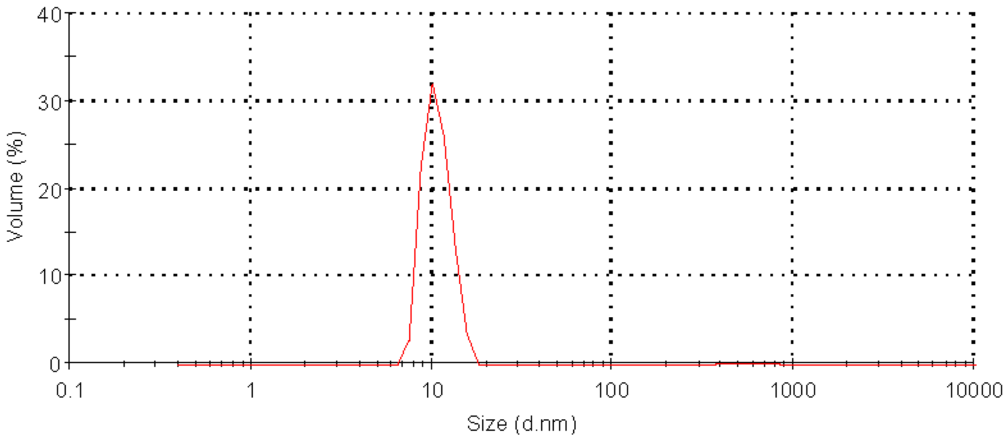

Figure S5: DLS of 100 µl PCR mixture using non-branched primers.

B) Y-shaped DNA construct: particle size 67 nm and 587 nm, polarization 90°, 10 measurements in 60 sec at 25 °C, 100 µL PCR mixture

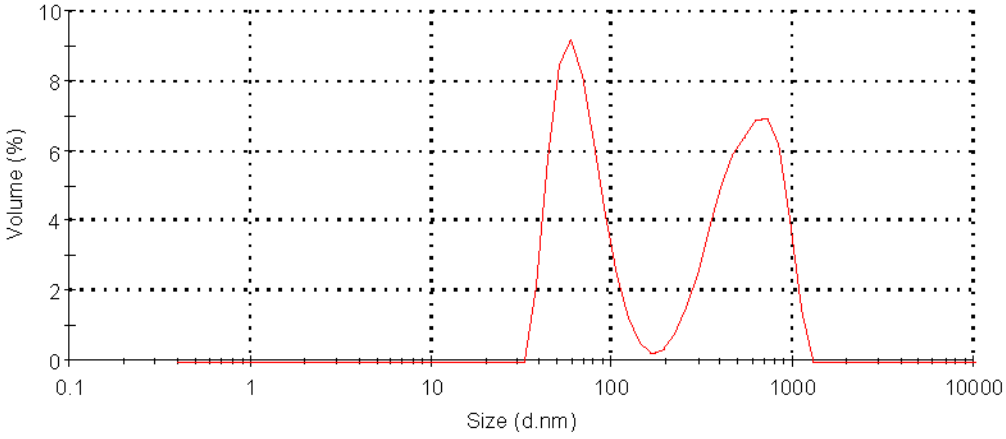

Figure S6: DLS of 100 µL PCR mixture using **ODN I** and **ODN II**.

Sample preparation for EPR Experiments

The modified Triphosphate dT*TP was synthesized as described in:

Obeid, S.; Yulikov, M.; Jeschke, G.; Marx, A. *Angew. Chem. Int. Ed.* **2008**, *47*, 6782-6785.

In the following PCR reactions a triethylammonium salt of dT*TP was used:

PCR was performed using a PCR-Thermocycler system (BIOMETRA). The reactions were performed containing 4 nM of the 1062 bp templates, 0.5 U of *Taq* DNA polymerase (Fermentas), 1× *Taq*-reaction buffer (75 mM TrisHCl (pH 8.8), 20 mM KCl and 0.01% Tween), 3 mM $MgCl_2$ and water were mixed in an overall volume of 25 μL. The final mixtures contained dNTPs (200 μM each of dATP, dGTP, dCTP and indicated ratios of dTTP to dT*TP), primers (0.5 μM each of the respective primer probe and reverse primer), 0.5 U *Taq* DNA polymerase (Fermentas; units defined by the supplier). PCR amplifications were performed by employing the following program: initial denaturation at 95 °C for 3 min, followed by 30 cycles of denaturation at 95 °C for 30 s, primer annealing at 60 °C for 90 s and extension at 72 °C for 60 s. PCR samples were purified using the following ethanol precipitation protocol: 25 μL PCR reaction mixture was mixed with 12.5 μL NH_4Ac (pH 5.5, 7.5 M) and 65 μL cooled, absolute EtOH. Mixture was stored at −20°C for 30 min and centrifuged at 13000 rpm at 4 °C for 30 min. The recovered DNA pellet was washed gently twice with cold 70% EtOH and the centrifuge step was repeated for each washing step. The recovered DNA was air dried and dissolved in 10 mM Tris-HCl (pH 8.5).

PCR using dT*TP

A) PCR with 1062 bp long template and linear primers

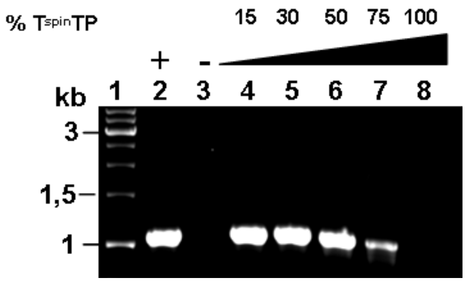

Figure S7: An PCR with linear primers and 1062 bp template using different ratios of dT*TP/dTTP: Lane 1: Ladder Generuler 1kb (Fermantas); Lane 2: PCR with all natural dNTPs; Lane 3: PCR with dATP, dGTP, dCTP; Lane 4, 5, 6, 7, 8: PCR with dATP, dGTP, dCTP and increasing ratios of dT*TP (15, 30, 50, 75, and 100 % of dT*TP, respectively).

B) PCR with branched primers **ODN I** and **ODN II** and 1062 bp template using different ratios of dT*TP/dTTP

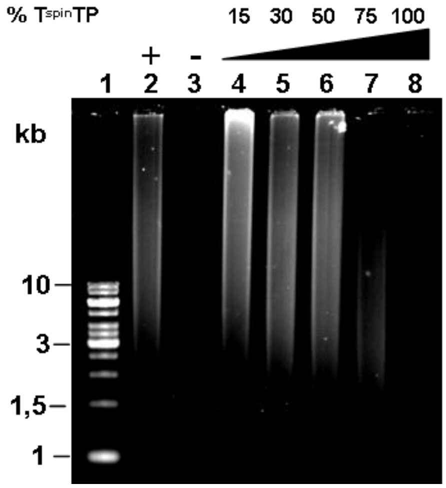

Figure S8: PCR with branched primers **ODN I** and **ODN II** and 1062 bp template using different ratios of dT*TP/dTTP: Lane 1: Ladder Generuler 1kb (Fermantas); Lane 2: PCR with all natural dNTPs, Lane 3: PCR with dATP, dGTP, dCTP; Lane 4, 5, 6, 7, 8: PCR with dATP, dGTP, dCTP and increasing ratios of dT*TP (15, 30, 50, 75, and 100 % of dT*TP, respectively).

EPR measurements

All spectra were recorded at T = 25 °C using a Bruker Elexsys E580 X-band spectrometer fitted with an Elexsys Super High Sensitivity Probehead (Bruker BioSpin GmbH) and a temperature controller (ITC503S, Oxford Instruments). Samples were loaded into glass capillaries (outer diameter 1 mm), with typical sample volumes of 10 μL. Performing field sweeps containing 4096 data points (in case of spectra from DNA networks 1024 data points) a modulation frequency of 100 kHz was used. Spectra were obtained with modulation amplitude of 0.4 G, microwave attenuation 20 dB, a sweep width of 80 G, and an accumulation time of 2 h.

In order to simulate the spectra, Matlab R2007b (The MathWorks, Inc., 3 Apple Hill Drive, Natick, MA 01760-2098, USA) and the toolbox EasySpin 3.1.6 were used. Varying simulation parameters, least-square-fits to experimental data were performed. For the simulations described in the present work A_{xx} = A_{yy} = 18.3 MHz, A_{zz} = 98.5 MHz and g = [gx gy gz] = [2.009 2.006 2.0024] were chosen. Error margins were determined by manually changing the parameters and to test in which range acceptable simulations of the data were obtained.

Table S1: Rotational correlation times as derived by spectral simulations of the EPR data.

	τ_{corr} [ns]	
**dT*TP**	0.09 ± 0.01	
PCR product resulting from non-branched primers in the presence of 50% dT*TP	0.15 ± 0.02	
	$\tau_{corr(1)}$ [ns]	$\tau_{corr(2)}$ [ns]
DNA networks generated by PCR in the presence of 50% dT*TP using branched primer as well as corresponding fraction	0.17 ± 0.02<br><br>8%	11.2 ± 0.3<br><br>92%
